# Energy Management Design of Dual-Motor System for Electric Vehicles Using Whale Optimization Algorithm

**DOI:** 10.3390/s25144317

**Published:** 2025-07-10

**Authors:** Chien-Hsun Wu, Chieh-Lin Tsai, Jie-Ming Yang

**Affiliations:** Department of Vehicle Engineering, National Taipei University of Technology, Taipei 10608, Taiwan; t113448035@ntut.org.tw (C.-L.T.); t111448026@ntut.org.tw (J.-M.Y.)

**Keywords:** whale optimization algorithm, electric vehicle, dual-motor, global grid search, hardware-in-the-loop

## Abstract

Dual-motor electric vehicles enhance power performance and overall output capabilities by enabling the real-time control of the torque distribution between the front and rear wheels, thereby improving handling, stability, and safety. In addition to increased energy efficiency, a dual-motor system provides redundancy: if one motor fails, the other can still supply partial power, further enhancing driving safety. This study aimed to optimize the energy management strategies of the front- and rear-axis motors, examining the application effects of rule-based control (RBC), global grid search (GGS), and the whale optimization algorithm (WOA). A simulation platform based on MATLAB/Simulink^®^ (R2021b, MATLAB, Natick, MA, USA) was constructed and validated through hardware-in-the-loop (HIL) testing to ensure the authenticity and reliability of the simulation results. Detailed tests and analyses of the dual-motor system were conducted under FTP-75 driving cycles. Compared to the RBC strategy, GGS and WOA achieved energy efficiency improvements of 9.1% and 8.9%, respectively, in the pure simulation, and 4.2% and 3.8%, respectively, in the HIL simulation. Compared to the pure RBC strategy, the RBC and GGS strategies incorporating regenerative braking achieved energy efficiency improvements of 26.1% and 29.4%, respectively, in the HIL simulation. Overall, GGS and WOA each present distinct advantages, with WOA emerging as a highly promising alternative energy management strategy. Future research should further explore WOA applications to enhance energy savings in real-world vehicle operations.

## 1. Introduction

In the face of accelerating technological advancement, scientists are actively developing innovative energy solutions to address climate change and resource depletion [1]. Rapid industrialization and population growth have intensified urban development, creating challenges in achieving sustainable and livable environments [2]. Transportation, as a core component of modern society, significantly influences both economic efficiency and sustainable development [3]. While automobiles, airplanes, and trains provide essential mobility, their growing energy demands have led to increased greenhouse gas emissions and concerns over depleting resources [4,5]. This escalating energy demand has prompted nations to reevaluate their energy strategies, promote environmental technologies, and pursue sustainable energy use [6]. Key efforts include expanding renewable energy, improving energy efficiency, and advancing green transportation technologies [7]. As a result, energy management in the transportation sector has become a focal point of innovation and sustainability [8]. Future vehicle development will concentrate on battery electric vehicles (BEVs), hybrid electric vehicles (HEVs), and hydrogen fuel cell electric vehicles (FCEVs) [9], with BEVs emerging as a major global focus. In response, automotive manufacturers are shifting toward new energy technologies, reshaping industry competitiveness and demonstrating a commitment to sustainability [10]. The transition from internal combustion engines to hybrid systems marks a key trend, with HEVs combining engines and electric motors for dual power output [11,12]. These systems adapt to varying conditions, improving performance, extending the range, and reducing fuel consumption, bringing them closer to the goals of clean mobility [13]. The optimization of energy and power systems focuses on energy conservation and cost reduction. Energy management strategies are central to this goal, enabling the efficient use of multiple energy sources and reducing environmental impacts [14]. In parallel, power system design—including component selection and the system architecture—ensures optimal system coordination and performance [15,16].

The power systems of BEVs can be divided into two main configurations: (1) single-motor drive and (2) multi-motor drive. The single-motor configuration features a simplified structure and the use of high-performance electric motors, which makes it more challenging to balance vehicle performance with improved energy efficiency [17]. Multi-motor drive systems can be further categorized into two types based on the number of motors: (1) four-motor distributed drive and (2) dual-motor coupled powertrain [18]. The four-motor distributed drive system features individual motors assigned to each wheel, with each wheel delivering power independently through separate reducers or drive shafts, or, alternatively, integrating motors directly into the wheels as hub motors [19]. In contrast, the dual-motor coupled powertrain employs two motors to achieve multiple driving modes. Through an ingeniously designed mechanical coupling mechanism, the system efficiently allocates power between the two motors, significantly enhancing motor utilization. Compared to conventional single-motor setups, this configuration achieves superior overall performance [20]. Therefore, the development of dual-motor electric vehicles not only enhances overall vehicle performance and operational flexibility but also contributes to the realization of high-efficiency, low-emission transportation solutions. It plays a crucial role in advancing green mobility and the development of intelligent vehicle technologies.

HEVs and dual-motor electric vehicles exhibit significant differences in their power distribution control strategies, primarily in terms of energy sources, control objectives, and the complexity of strategies. Specifically, HEVs convert motor electricity consumption into an equivalent fuel consumption measure, whereas dual-motor electric vehicles calculate the minimum motor electricity consumption directly. Despite these differences, the two vehicle types share similarities in their energy distribution control methodologies, allowing for the application of analogous power distribution control strategies. According to previous studies, the optimization of a vehicle’s energy management system primarily aims at reducing the overall energy consumption and lowering the costs to enhance the overall efficiency and system performance. Energy management system strategies can mainly be categorized into two types: (1) rule-based (RB) strategies and (2) optimization-based strategies [21]. Rule-based strategies can be further divided into two main types: (1) deterministic rules and (2) fuzzy logic rules [22]. Deterministic rules involve predefined control strategies based on engineering experience to manage switching between different operational modes of the powertrain and distributing power among various energy sources [23]. In contrast, fuzzy logic rules employ a set of predetermined yet flexible control strategies, allowing the more adaptable handling of uncertainties and complexities within the system. Decisions and controls are executed through fuzzy logic, enabling dynamic adjustments to operational modes and power allocation between different energy sources [24]. Optimization-based strategies can be mainly classified into two categories: (1) real-time optimization and (2) global optimization [25]. Real-time optimization involves dynamically allocating power between the engine and electric motor based on the current power demands, ensuring the minimum equivalent consumption and minimal power losses at each moment. An example of this approach is global grid search (GGS) [26]. Global optimization utilizes optimal control principles to improve fuel consumption and power loss across an entire driving cycle, dynamically optimizing power allocation among energy sources to achieve overall optimal vehicle performance. Examples include methods such as dynamic programming (DP) [27] and genetic algorithms (GAs) [28]. To thoroughly investigate vehicle energy efficiency, this research primarily focuses on two main areas: the powertrain system and the control strategy. Regarding powertrain design, recent commercially available vehicles exhibit highly similar system architectures, with multi-power-source configurations emerging as the mainstream design. In terms of control strategies, theory-based controls have already achieved considerable success in various engineering applications. Currently, bio-inspired heuristic algorithms, such as the whale optimization algorithm (WOA), are gradually becoming a popular trend in the field of optimization methods [29]. The WOA, compared with other heuristic algorithms, demonstrates superior problem-solving capabilities and computational efficiency. It is feasible for energy management involving multiple control variables, thereby enhancing the performance of hybrid power systems and showcasing its significant potential in vehicle control applications [30]. In addition, hierarchical reinforcement learning (HRL) algorithms can be effectively applied to vehicle energy management. Specifically, in dual-motor or hybrid electric vehicles, HRL can determine overarching vehicle power objectives, such as energy-saving mode or power mode, while simultaneously managing detailed controls for individual motors or power sources. These detailed controls include the front and rear motor torque distribution, generator operation, and battery charge–discharge management. By employing hierarchical management, HRL significantly reduces the complexity involved in designing vehicle control strategies, thereby enabling the rapid and efficient determination of optimal control solutions [31].

To effectively validate the practical operation of the energy management system, a HIL system is utilized to perform real-time computation, bridging the gap between the simulation phase and actual vehicle implementation. This approach helps to identify data errors and enhances the system’s fault tolerance. In this research, the dual-motor drive simulation platform is divided into two parts: the vehicle control unit (VCU) and the vehicle platform. An Arduino DUE microcontroller and a Ti C2000 microcontroller are employed to construct a hardware-in-the-loop (HIL) platform, enabling analog and digital input/output signal conversions through signal processing. The Federal Test Procedure 75 (FTP-75) driving cycle is used as a basis for the analysis of the performance characteristics and inter-system response relationships, including the torque, rotational speed, optimization management, and overall vehicle energy efficiency.

## 2. Establishment of a Vehicle Dynamics Model

### 2.1. Electric Vehicle Simulation Platform

**Figure 1:**
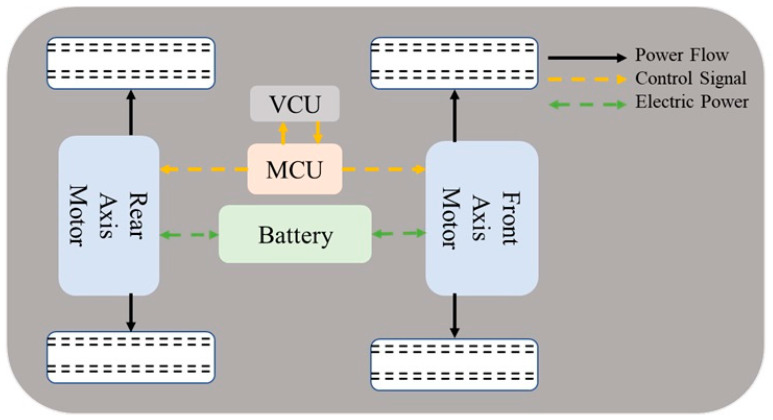
System architecture of the dual-motor drive system.

The objective of this study is to establish a dual-motor drive system, using a high-chassis Tesla Model X as the base vehicle specification [32]. The vehicle is equipped with a 95 kW ZEPT drive motor integrated with a gearbox, provided by ZEPT (Zero-Emission Power Train), a company based in Taoyuan City, Taiwan, specializing in complete electrification solutions for mobile vehicles. The motor specifications are proportionally scaled according to the power requirements [33]. Finally, the power distribution is optimized through control strategies, as shown in Table 1. This study adopts a dual-motor drive system architecture, with the vehicle system featuring an independent suspension design for both the front and rear axles. The model is based on test data from the dual motors and lithium battery pack of the actual vehicle model (Tesla Model X). The detailed structure is shown in Figure 1.

**Figure 2:**
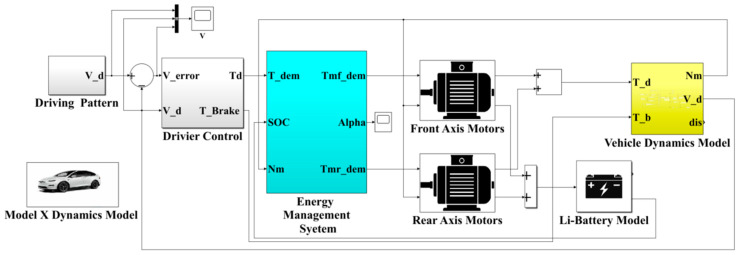
Schematic diagram of the vehicle simulation platform software.

This study focuses on the optimization of energy management strategies to evaluate the rationality of motor power distribution and performance data through comparative analysis. The drive motors transmit power to the vehicle’s wheels via gearboxes, and a vehicle velocity tracking simulation model is used for detailed analysis. First, the velocity error is calculated as the difference between the driving cycle velocity and the actual vehicle velocity. A PI controller is then used to compute the demanded vehicle torque, and the resulting signal is sent to the optimized energy management system. Considering the demanded vehicle velocity and the battery’s state of charge (SOC), an optimization algorithm is applied to determine the total demanded torque. In this study, the vehicle simulation platform is built using MATLAB/Simulink^®^ (R2021b, MATLAB, Natick, MA, USA), as shown in Figure 2. The platform includes the following models: driving cycle, driver, motor, battery, vehicle dynamics, and energy management system. The FTP-75 driving cycle adopted in this study was initially established as a standard for the measurement of the exhaust emissions and fuel consumption of gasoline vehicles in the United States. Currently, it is also utilized to evaluate energy efficiency in EVs. FTP-75 primarily assesses EVs’ energy consumption characteristics, including electric energy usage and driving range performance, in both low-speed urban conditions and high-speed scenarios. The driving cycle incorporates multiple acceleration and deceleration events, simulating the frequent stop-and-go traffic typical of urban environments, thereby enabling a rigorous evaluation of the torque distribution performance in dual-motor configurations under demanding conditions. As shown in Figure 2, due to the rapid responses of electric motors, with a reaction time of approximately 0.1 s, the required torque from the front- and rear-axle motors can be summed to yield the total demand torque. This approach indicates that both motors effectively contribute torque in the same direction to propel the vehicle forward. Subsequently, the physical constraints and motor efficiencies will be computed through detailed motor models.

### 2.2. Driving Cycle Model

**Figure 3:**
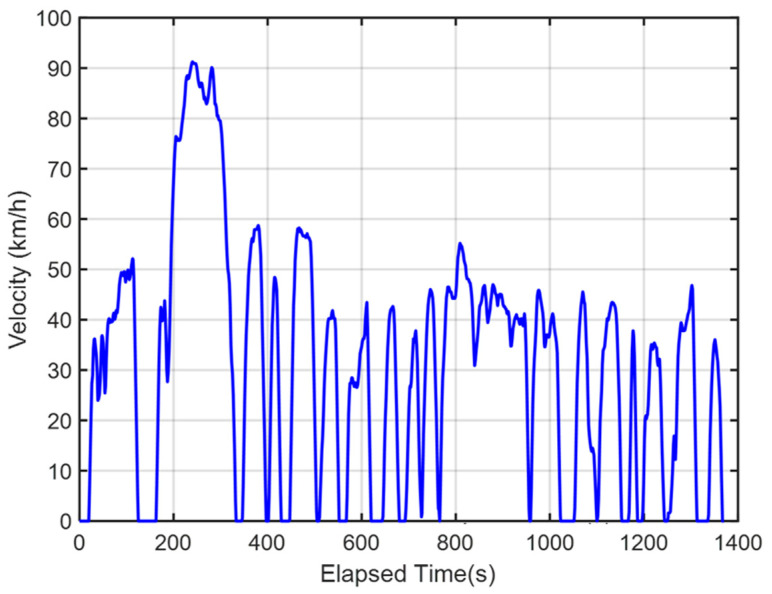
FTP-75 driving cycle.

FTP-75 is a standardized testing procedure mandated by the U.S. Environmental Protection Agency (EPA) for the evaluation of vehicle fuel efficiency. It simulates both urban and highway driving conditions and uses these conditions to calculate a vehicle’s fuel consumption and emission data [34]. The FTP-75 test procedure includes a series of predetermined driving cycles, velocities, and idle periods to reflect the vehicle’s performance in real-world usage scenarios. In this study, the FTP-75 test cycle with a total driving time of 1370 s and a maximum velocity of approximately 90 km/h is adopted, as shown in Figure 3.

### 2.3. Driver Model

Proportional–integral (PI) control is a commonly used type of control system in engineering. It combines proportional control (P) and integral control (I) to improve the response velocity and accuracy of the control system, resulting in better overall performance [35]. By integrating both proportional and integral components, the controller can respond quickly to error changes while eliminating steady-state errors, ensuring that the final output reaches the demanded set point. In the vehicle simulation, the actual vehicle velocity and the demanded velocity are calculated and subtracted to obtain the velocity error, which is then used as input to the driver model. The output of the PI controller provides the total demanded torque and braking torque to appropriately adjust the vehicle’s demanded power, as expressed by the following Equation (1):

(1) $$T_{d}\left(t\right)=K_{P}\cdot V_{err}(t)+K_{I}\cdot \int V_{err}(t)$$

where $T_{d}$ is the total demanded torque; $K_{P}$ is the gain constant of proportional control; $K_{I}$ is the gain constant of integral control; $V_{err}$ is the velocity error.

### 2.4. Drive Motor Model

**Figure 4:**
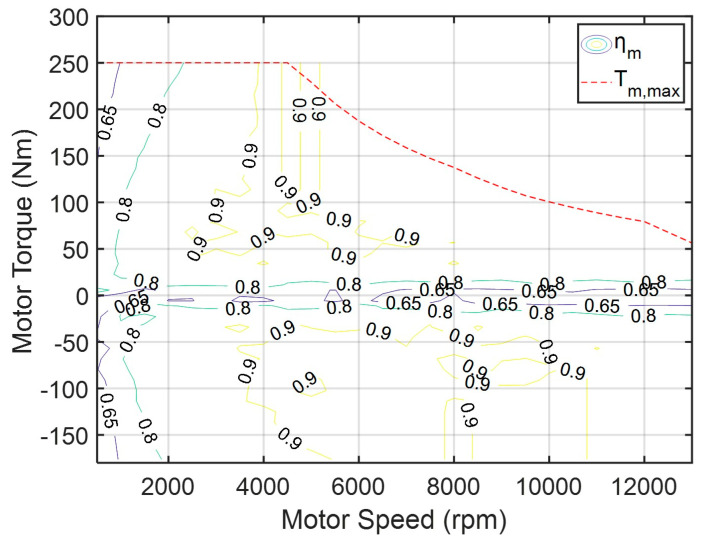
Efficiency curve of the drive motor.

In this study, the drive motor model receives the demanded drive torque output from the driver model, considering the motor’s maximum physical speed limitation; computes the feasible torque; and delivers the appropriate drive torque for vehicle propulsion. The mechanical efficiency curve of the drive motor is shown in Figure 4. The motor efficiency is determined using a two-dimensional lookup table based on the current motor speed and torque. The efficiency calculation is represented by Equation (2):

(2) $$\eta_{m}\left(t\right)=f(T_{m}\left(t\right),\;N_{m}(t))$$

where $\eta_{m}$ is the efficiency of the drive motor; $T_{m}$ is the output torque of the drive motor; $N_{m}$ is the speed of the drive motor.

### 2.5. Energy Storage Battery Model

The lithium battery serves as the primary power source for the vehicle’s drive motors. Based on real-world data obtained from the lithium battery, a relationship was established between the internal resistance and voltage, which can be used to estimate the battery’s SOC. In the model developed for this study, the battery temperature is assumed to be constant and does not vary with the operating conditions or duration. The accuracy of the battery’s SOC estimation is critical, as it significantly influences the vehicle’s dynamic performance and is one of the key parameters in energy management strategies. This study adopts an internal resistance model, in which the battery contains an equivalent internal resistance value $R_{b}$, which varies with the battery’s ${SOC}_{b}$ and temperature $t_{b}$, as expressed in Equation (3):

(3) $$R_{b}=R_{b}\left({SOC}_{b},{\;t}_{b}\right)$$

where $R_{b}$ is the internal resistance value of the battery; ${SOC}_{b}$ is the battery’s state of charge; $t_{b}$ is the temperature of the battery.

The variation in the battery state exhibits a first-order dynamic response. The SOC ranges from 0 to 1 (0–100%). This relationship is expressed by Equation (4):

(4) $${SOC}_{b}=\frac{{SOC}_{int}\times Q_{rated}-\int \frac{I_{b}}{3600\times \eta_{b}}dt}{Q_{rated}}$$

where $Q_{rated}$ is the rated capacity of the lithium battery; $I_{b}$ is the discharge current of the battery; $\eta_{b}$ is the charge–discharge efficiency of the battery. $\eta_{b}$ is a function of the relationship between $V_{OC}$ and $I_{b}$:

(5) $$\eta_{b}=\eta_{b}\left(V_{OC},I_{b}\right)$$

The discharge current of the battery calculation method is as shown in Equation (6):

(6) $$I_{b}=\frac{V_{OC}-\sqrt{{V_{OC}}^{2}-4\times P_{b}\times R_{b}}}{2\times R_{b}}$$

where $V_{OC}$ is the open-circuit voltage of the battery, a function of ${SOC}_{b}$ and $I_{b}$; $P_{b}$ is the discharge power of the battery to the drive motor. The calculation method for the load voltage of the battery during discharge is shown in Equation (7):

(7) $$V_{b}=V_{OC}-I_{b}\times R_{b}$$

The dynamic performance of the battery resistance is calculated using the internal resistance method. Based on experimental data from individual energy storage cells, the variation in internal resistance under different SOC conditions can be estimated. This allows for the determination of the open-circuit voltage (OCV) values corresponding to different SOCs.

### 2.6. Vehicle Dynamics Model

The vehicle dynamics model is a critical component of the entire system, used to simulate the vehicle’s dynamic behavior under various driving conditions. When analyzing driving scenarios, it is essential to consider various forms of resistance. The vehicle’s traction force is equal to the sum of the forces acting in the direction opposite to its motion. During operation, the vehicle primarily encounters three types of resistive forces: air resistance, rolling resistance, and gradient resistance. These forces are closely related to the vehicle’s performance and operation. The vehicle’s acceleration force is equal to the driving force minus the air resistance, gradient resistance, rolling resistance, and braking force; these forces can be expressed as in Equation (8):

(8) $$m_{EV}\frac{dV_{v}}{dt}=\frac{T_{f}\eta_{f}}{R_{w}}-\frac{1}{2}\rho_{a}C_{d}A_{f}V_{v}^{2}-\mu m_{EV}g\cos\left(\theta \right)-m_{EV}g\sin\left(\theta \right)-F_{brk}$$

where $C_{d}$ is the drag coefficient; $A_{f}$ is the frontal area of the electric bus; $\rho_{a}$ is the air density; $V_{v}$ is the vehicle velocity; $m_{EV}$ is the total vehicle mass; μ is the rolling resistance coefficient; g is the gravitational acceleration; θ is the gradient angle; $T_{f}$ is the output torque of the final drive; $\eta_{f}$ is the overall efficiency of the final drive; $F_{brk}$ is the braking force.

### 2.7. Hardware-in-the-Loop System Architecture

In the development process, the HIL system plays a critical role as an indispensable tool to ensure that the product operates smoothly before entering mass production. Through this system, the functionality and feasibility of the controller can be verified in advance to assess whether it meets the expected performance [36]. This helps to identify potential issues early, allowing for timely corrections that reduce the cost of later-stage problem-solving, while also ensuring product safety and functional reliability. It also allows the observation of whether the output results exhibit distortions or delays that need to be addressed. This step is essential in the phase prior to technological development and commercialization, facilitating faster and more accurate debugging [37]. In this study, the HIL system platform was built using an Arduino DUE microcontroller and a TI C2000 microcontroller. Both the real-time model of the full vehicle platform and the real-time model of the vehicle controller were developed using the MATLAB/Simulink^®^ software. These models simulate the operation of the entire vehicle system in real time and allow for immediate computation and validation to ensure optimal vehicle performance under various conditions. The driving cycle provides the demanded vehicle velocity to the driver model. The PI controller then outputs the demanded vehicle torque. Simultaneously, the battery model calculates the battery’s SOC, and the vehicle dynamics model computes the motor output speed. These three outputs are transmitted to the receiving end of the VCU. Through the energy management strategy, power is distributed to the front-axis motor and rear-axis motor and then sent back to the receiving end in the vehicle system. During this signal transmission process between the two models, the system continuously converts the vehicle’s digital signals into voltage signals and then converts these voltage signals back into the digital signals required by the vehicle, ensuring smooth communication between the two systems at all times.

**Figure 5:**
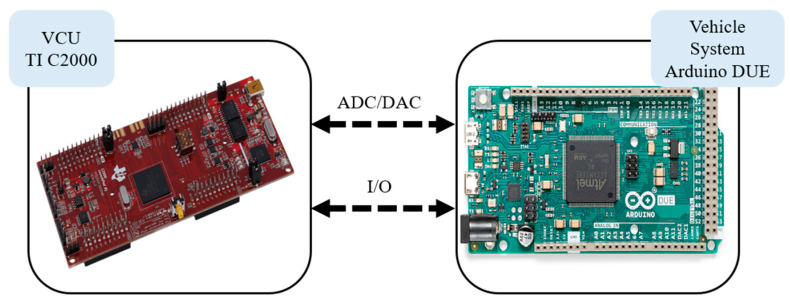
System architecture of the HIL using the Arduino DUE and Ti C2000.

In this study, the Ti C2000 microcontroller is connected to the Arduino DUE microcontroller to perform HIL system testing. The HIL system architecture is shown in Figure 5. To verify the authenticity of the dual-motor drive power system, the setup is divided into two parts: the real-time model of the vehicle platform and the real-time model of the VCU. This division allows for better control and testing of each component of the system, ensuring performance and reliability under real-world applications. Before performing signal processing, it is necessary to understand how analog signals are converted into voltage by the Arduino DUE and Ti C2000. The Arduino DUE converts analog signals ranging from 0.56 to 2.76 V into digital values ranging from 0 to 4095, while the Ti C2000 converts analog signals from 0 to 3.3 V into the same digital range, 0 to 4095. The Arduino DUE is a feature-rich microcontroller, whereas the Ti C2000 series is a high-performance microcontroller designed specifically for control applications. Due to the differences in voltage handling between the two microcontrollers, proportional amplification or attenuation is required, as shown in Table 2. The system’s pin assignments include the demanded torque, battery SOC, and target speed. These are output from the Arduino DUE and, after signal conversion, serve as input to the Ti C2000. The Ti C2000 then performs optimized calculations based on the energy management strategy and outputs the demanded signals, including the motor power distribution ratio, the front demanded motor torque, and the rear demanded motor torque. These signals are then converted and sent back to the Arduino DUE as input signals, as summarized in Table 2. Therefore, the digital and analog input/output signals between the HIL system and the controller are defined, establishing a communication frequency and timing synchronization at 1 ms intervals. Testing conditions are then configured, utilizing the FTP-75 driving cycle as the operational input scenario. The monitored indicators include the front and rear motor torque variations, total energy consumption, torque distribution ratios, system response time, and operational stability. Multiple test runs are conducted to verify the consistency and reliability of the implemented algorithm. Compared to traditional pure simulation methods, which enable the rapid verification of feasibility but cannot accurately reproduce hardware delays, communication interference, or non-ideal behavior, the HIL technique provides a realistic and physically interactive testing environment. This allows energy management systems to be evaluated under conditions closely resembling real-world vehicle operations, significantly enhancing the practicality and reliability of control strategies. Through HIL testing, this study not only verifies the practicality of various strategies but also assesses the fault tolerance under simulated failure scenarios, effectively bridging the gap between purely simulated and real-world testing. Consequently, HIL plays a critical role in verifying energy management systems, effectively mitigating deployment risks before system implementation.

## 3. Energy Management Strategy

**Figure 6:**
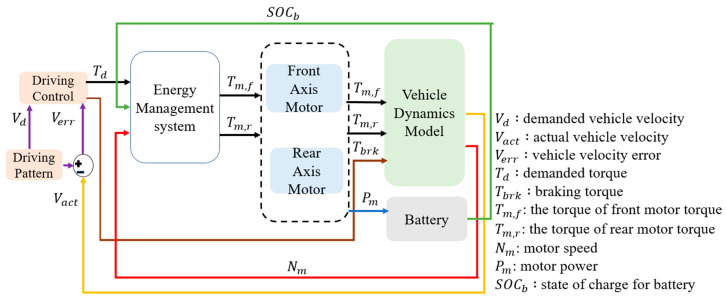
Control architecture of the energy management system.

This study adopts an energy management strategy to calculate the optimal motor efficiency range and determine the best power distribution ratio. Simulation verification is conducted using the FTP-75 driving cycle, and the control architecture of the energy management system is illustrated in Figure 6. First, the driving cycle model provides the target vehicle velocity for simulation. The actual vehicle velocity is then calculated through the vehicle dynamics model. The vehicle velocity error, obtained by subtracting the actual velocity from the demanded vehicle velocity, is used as the input to the PI controller. The PI controller outputs the demanded torque, which is sent to the energy management model to determine the power distribution between the dual drive motors. Meanwhile, the braking torque is used to command the braking force and is sent to the vehicle dynamics model to perform vehicle braking. The energy management model uses the actual motor speed from the vehicle dynamics model to calculate the optimal torque distribution, sending control commands to the front- and rear-axis motors for execution. Finally, the motor outputs are integrated into the vehicle dynamics model to compute the actual vehicle velocity.

The energy management system dynamically allocates the output torque of the front- and rear-axle motors based on the current driving conditions, summing their contributions to generate the total driving torque. However, the actual wheel driving force is primarily determined by the vehicle’s load conditions, including the acceleration demand, rolling resistance, and aerodynamic drag. The total driving torque is fed back to the control system through the vehicle dynamics module, forming a closed-loop control architecture that enables real-time adjustment and energy optimization. Consequently, this torque distribution strategy directly influences the overall energy efficiency. Although the front- and rear-axle motors are of identical specifications, their operating speeds and output torques differ during actual operation, resulting in variations in their individual energy conversion efficiencies. Therefore, by dynamically adjusting the torque split parameter (α) within the energy management system, it is possible to optimize the operating points of each motor according to their efficiency maps. This approach minimizes the total energy consumption and extends the driving range. In addition, the model incorporates a vehicle dynamics module to calculate the required wheel driving force under different operating modes. As the load increases, the system can dynamically modify the torque distribution between the front and rear axles to enhance the traction performance and driving response. Thus, even when using motors of the same specifications, an appropriate torque distribution strategy can achieve an optimal energy efficiency and performance balance under various driving conditions. This demonstrates that the proposed distribution strategy in this study holds practical significance and real-world applicability.

### 3.1. Rule-Based Control Strategy

Rule-based control (RBC) is implemented using the Stateflow model in MATLAB/Simulink^®^ to develop the energy management strategy. Based on different driving demands, corresponding power distribution modes are established, as detailed in Table 3. When the input signals meet the predefined state conditions, the system outputs the corresponding power distribution results. These rules are primarily formulated based on the researcher’s engineering experience and the operational efficiency of individual components, enabling control over mode switching under various driving conditions [38]. In this study, the RBC strategy defines four operating modes, as shown in Table 3. The mode determination is based on the demanded vehicle velocity, demanded torque, and battery SOC. The modes are defined as follows.

➢ System Ready Mode: When the driver’s demanded torque is 0 Nm, the system enters the ready mode, and the commanded motor torque output is also set to 0 Nm.
➢ Low-Load Mode: When the demanded torque is greater than or equal to 0.1 Nm, the vehicle enters the low-load mode. In this mode, the demanded torque is shared between the front- and rear-axis motors with a distribution ratio of 3:7.
➢ High-Load Mode: When the actual vehicle velocity exceeds 50 km/h, the system switches to high-load mode. In this mode, the demanded torque is allocated to the rear-axis motor, which primarily propels the vehicle.
➢ Safety Mode: When the demanded torque is 0 Nm and the battery SOC reaches 0, the system enters the safety mode.
➢ These control rules ensure appropriate power allocation and system responses under various operating conditions.

Since dual-motor electric vehicles share a single battery system, the system will automatically enter a protection mode and reduce the power output when the battery SOC is low. This study focuses on the torque distribution between the front and rear motors under normal operating conditions, based on the actual vehicle speed, and therefore does not take the battery SOC into account. An SOC value of zero indicates either a fully depleted battery (activating the battery’s built-in protection mechanism) or a disconnection, both of which trigger system safety protocols. These conditions must be considered in practical applications.

### 3.2. Global Grid Search Method

In the computational process, system model parameters such as the total demanded power, motor speed, and physical constraints are established to construct an optimized power distribution model for control system parameter analysis. This approach effectively simplifies the control model to meet the real-time computing requirements of the HIL system. To obtain the optimal control model parameters, a GGS method is used to find the best parameter solution. By first inputting the necessary parameters, such as the demanded torque and drive motor speed, and performing discretization, an objective function is defined to identify the optimal power distribution algorithm. The goal is to minimize the power consumption, and the minimum value is determined as expressed in Equation (9):

(9) $$J=min({\dot{E}}_{m,f}+{\dot{E}}_{m,r}+\omega )$$

where $\eta_{m,f}$ is the efficiency of the front-axis motor; $\eta_{m,r}$ is the efficiency of the rear-axis motor; ${\dot{E}}_{m,f}$ is the input power of the front-axis motor; ${\dot{E}}_{m,r}$ is the input power of the rear-axis motor; J is the optimal cost function; and ω is a penalty value. The output powers of the front- and rear-axle motors are calculated as shown in Equations (10) and (11):

(10) $${\dot{E}}_{m,f}=\frac{T_{m,f}\times N_{m}}{\eta_{m,f}}$$

(11) $${\dot{E}}_{m,r}=\frac{T_{m,r}\times N_{m}}{\eta_{m,r}}$$

By substituting Equations (10) and (11), the result is as shown in Equation (12):

(12) $$J=\min(\frac{T_{m,f}\times N_{m}}{\eta_{m,f}}+\frac{T_{m,r}\times N_{m}}{\eta_{m,r}}+\omega )$$

Here, ω represents the penalty value; when the conditions of grid search exceed the physical constraints, a large penalty value is assigned:

(13) $$\omega =10^{6}$$

In studies related to the front and rear motor power ratio, it has been observed that, when electric vehicles require more acceleration and high-speed operation, the importance of the front-axle motor may increase [39,40]. The GGS method selects the optimal power distribution by minimizing the power consumption based on the vehicle’s real-time operating status and control variables, while also taking the vehicle performance requirements into account. This enables the vehicle to achieve the most efficient energy management at different operating points, thereby improving overall energy utilization. The strategy process is as follows:

➢ The GGS search method involves three nested loops used to globally search discretized values of the demanded torque and motor speed;
➢ The program uses “if–then–else” conditional statements to evaluate various possible operating modes and then calculates parameters such as the efficiency and torque of the front- and rear-axis motors;
➢ Based on the concept of minimum power consumption, the power consumption under different conditions is calculated—for a fixed motor speed and demanded torque, the minimum power consumption under different dual-motor torque distributions can be used to determine the optimal power distribution ratio.

Table 4 lists the relevant parameters for the front- and rear-axle motors. Based on the design of the optimal objective function, the power distribution ratios are defined according to Equations (14) and (15):

(14) $$\alpha =\frac{T_{m,f}}{T_{d}}$$

(15) $$\left(1-\alpha \right)=\frac{T_{m,r}}{T_{d}}$$

where α is the power distribution ratio (0~1).

**Figure 7:**
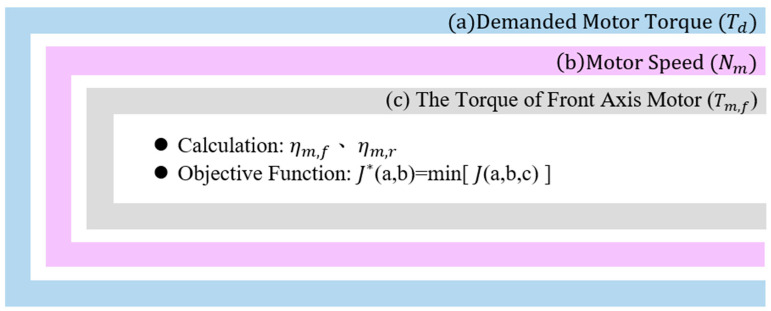
Optimal power distribution loop for the front- and rear-axis motors.

Under the same minimum motor power consumption strategy, the optimal motor power consumption is identified using the optimal power distribution loop shown in Figure 7. The discretized variables include the required torque, the motor speed, and the front- and rear-axle motor power distribution ratio α, which are used to perform a global search. By calculating the electric power consumption of the front- and rear-axis motors under different variable conditions, an optimal two-dimensional power distribution map can be derived. The calculation is expressed in Equation (16) and (17):

(16) $$\alpha =\frac{T_{m,f}}{T_{d}}$$

(17) $$\left(1-\alpha \right)=\frac{T_{m,r}}{T_{d}}$$

In the energy management system of this study, the demanded torque serves as the primary input parameter, determined by the accelerator pedal position, reflecting the driver’s demanded torque. The battery SOC affects the motor output torque, imposing limitations at lower SOC levels based on the battery capabilities. The rotational speeds of the front and rear motors are calculated according to the current vehicle velocity and the drivetrain characteristics, such as the tire radius and final gear ratio. Subsequently, a nested loop calculation is conducted, considering the motor speed range, torque limits, maximum torque capabilities, and motor efficiency maps, enabling the selection of an optimal operating point under all conditions to minimize the overall energy consumption and achieve global energy efficiency optimization. Consequently, this optimization procedure determines the torque distribution ratio between the front and rear motors.

### 3.3. Whale Optimization Algorithm

In this study, a heuristic optimization algorithm inspired by the foraging behavior of whales is utilized. The WOA simulates the process by which whales search for food, perceiving information such as sounds and scents in their surroundings to determine the direction and distance of prey and then taking corresponding actions. The algorithm mimics these behaviors to search for optimal solutions [41]. One of the most remarkable features of whales is their unique hunting method, known as the bubble-net feeding method. This feeding behavior typically occurs near the ocean surface, where whales create bubbles to trap prey. They form these bubble nets along circular or figure-of-eight-shaped paths. During the upward spiral, a whale dives to a depth of approximately 12 m and begins creating spiral-shaped bubbles around the prey, eventually swimming upward to capture it. The dual-spiral strategy involved in this behavior includes three distinct stages: spiral ascent, surface tail slapping, and the capture cycle. Bubble-net feeding is a unique and specialized behavior among whales, and the WOA is modeled after this spiral bubble-net hunting strategy to achieve optimization objectives [42]. The strategy process is as follows.

**Figure 8:**
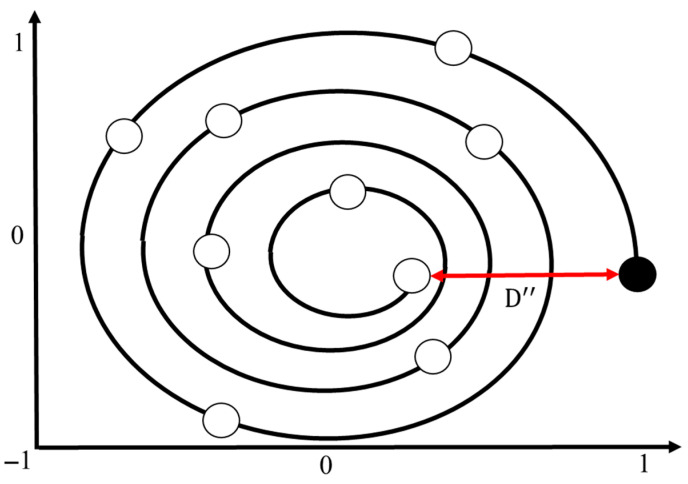
Update diagram of the spiral position.

**Figure 9:**
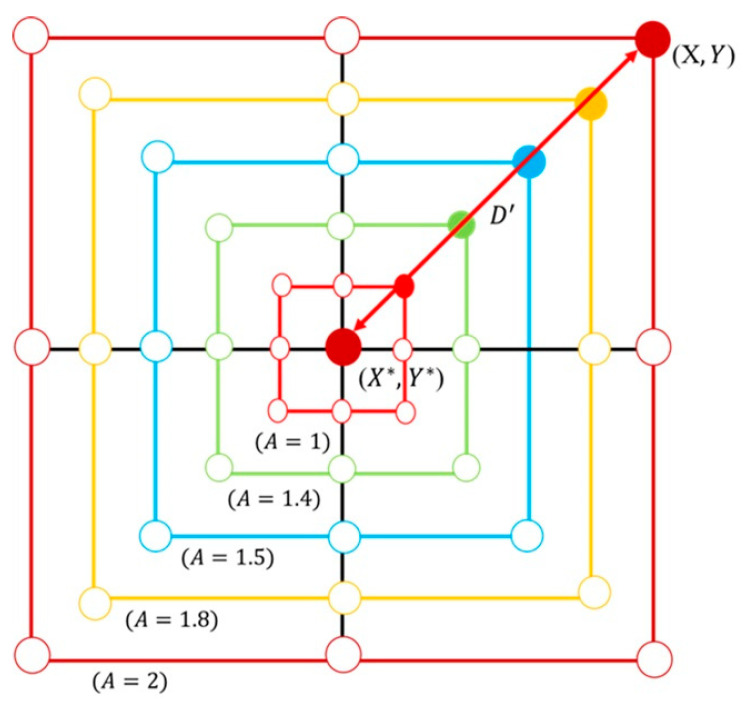
Search diagram of the whale prey.

(1) Initialization: In this stage, the initial parameters are set and the initial positions of the whales are generated. To prevent the algorithm from falling into local optima, the whales are uniformly distributed throughout the search space. The initial positions are defined by Equation (18):
  (18) $${Whales}_{position}=unifrnd({Low}_{bound},{Up}_{bound},{Variable}_{size})$$
  where ${Low}_{bound}$ is the lower bound of the control variable being searched; ${Up}_{bound}$ is the upper bound of the control variable being searched; ${Variable}_{size}$ is the dimensional size of the control variable.
(2) Surround prey: The whale identifies the position of the prey and encircles it. In the WOA, it is assumed that the current best solution is the target prey or is close to the optimal solution [43]. Once the best search formula is defined, other search agents will attempt to update their positions toward the best one. This behavior can be expressed by the following equation:
  (19) $$X(t+1)=X^{*}\left(t\right)-A\cdot D^{\prime \prime }$$
  (20) $$D^{\prime \prime }=|C\cdot X^{*}\left(t\right)-X(t)|$$
  where t is the current number of iterations; A is the step coefficient; C is the weighting coefficient; $D^{\prime \prime }$ is the spatial vector between the whale and the current best prey position; $X^{*}$ is the position of the current best solution; X is the current position. Whenever a better solution appears during each iteration, an update is required. A, a, and C are calculated as follows:
  (21) A=2a·r−a
  (22) $$a=2-n_{t}\cdot \frac{2}{n_{max}}$$
  (23) C=2·r
  where a decreases linearly from 2 to 0 during the iteration process; $n_{t}$ is the current iteration number; $n_{max}$ is the maximum number of iterations; r is a random vector within the range [0, 1].
(3) Bubble-net attacking method: There are two methods of modeling the whale’s bubble-net feeding behavior: (1) the shrinking surround mechanism and (2) the spiral position update [44]. As shown in Equation (21), this behavior is achieved by gradually decreasing the value of a. It is important to note that the range of A also decreases along with a, where A is a random value within the interval [−a, a]. By setting A as a random number between −1 and 1, the new position of a search agent can lie anywhere between its original location and the current best position. When 0≤A≤1, it illustrates all possible locations in the 2D space between (X, Y) and (X*, Y*), which essentially simulates the behavior of surrounding and hunting prey. Whales generate spiral-shaped bubble nets through their blowholes to trap prey, making it difficult for the prey to escape. They then move along this spiral bubble path to complete the hunt [45]. Based on the position of the best-identified prey, the whale generates a spiral bubble pattern and updates its position accordingly using Equation (23), as shown in Figure 8. The spiral position update is expressed by Equation (24):
  (24) $$X\left(t+1\right)=D^{\prime \prime }\cdot e^{bj}\cdot \cos2\pi j+X^{*}(t)$$
  where $D^{\prime \prime }$ is the spatial vector between the whale and the current best prey position; b is a constant that defines the shape of the spiral bubble net, and it is set to 1 in this study; j is a random number within [−1, 1].
(4) Search for prey: In addition to using the bubble-net method, whales also exhibit random prey-searching behavior during foraging, as illustrated in Figure 9. This behavior is based on a variable A vector, where whales perform random searches relative to each other’s positions. In this method, when A is greater than 1 or less than −1, it drives the search to move away from the current location. Unlike the bubble-net method, this search mechanism does not rely on the best solution found so far, but rather updates the positions based on randomly selected search agents [46]. This mechanism involves an A vector magnitude greater than 1, as represented in Equations (25) and (26):
  (25) $$X\left(t+1\right)=X_{rand}-A\cdot D^{\prime }$$
  (26) $$D^{\prime }=|C\cdot X_{rand}-X|$$
  where $X_{rand}$ is the current random position of the whale population; $D^{\prime }$ is the position vector between the whale and a randomly selected prey.
(5) Record the current highest profit until the search stopping condition is met: The WOA continuously updates the optimal solution through iterative searching (i.e., minimizing the objective function defined in this study). Once the search stopping condition is met, the algorithm outputs the optimal solution; otherwise, it returns to steps (2), (3), and (4) to continue the computation until the stopping condition is satisfied or the computation is complete [47].

This study introduces two optimization methods for control strategies. Although the core computations differ in terms of the mathematical equations used during their respective search processes, both aim to optimize the same objective function, and the control variables used are identical to those in the GGS approach, as referenced in the GGS power distribution ratio formula. In the WOA, key control parameters typically include the population size and the number of iterations. These parameters significantly affect the algorithm’s performance and convergence speed. In general, their values are adjusted according to the characteristics and requirements of the problem being addressed. In this study, vehicle energy efficiency simulations are conducted using MATLAB/Simulink^®^, with a sampling time of 0.01 s. The main focus is to compare the impacts of different control strategies on the vehicle energy efficiency, rather than on the optimization of the algorithm parameters themselves. Based on the simulation results, using a larger population size yields better outcomes. The parameter settings are shown in Table 5.

**Figure 10:**
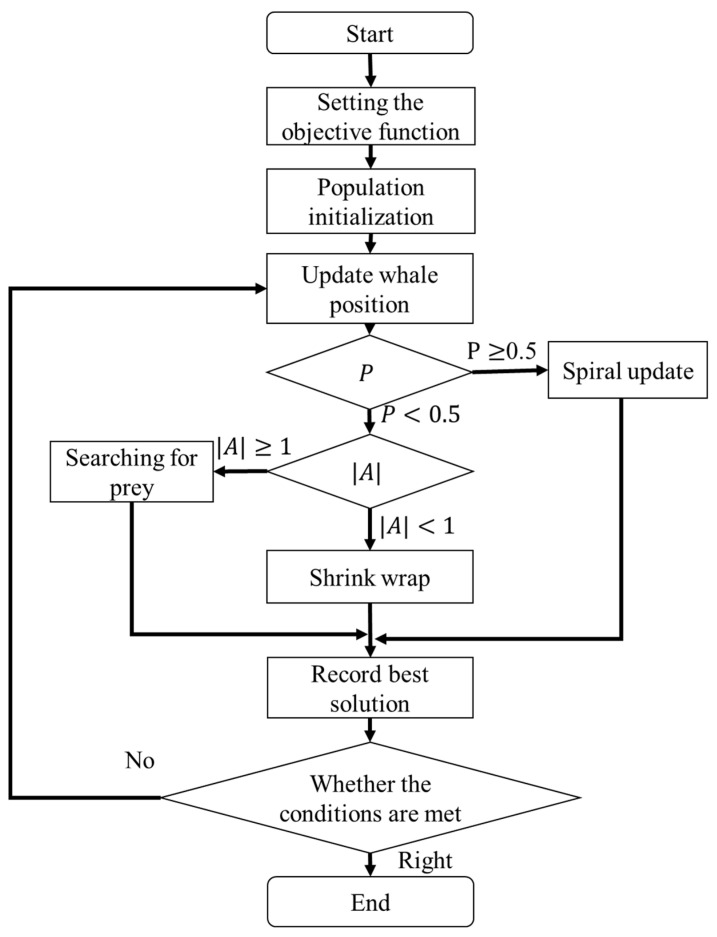
Flowchart of the WOA.

In this study, the defined parameters are applied to the WOA for computation. The process begins with Step 1, which involves initializing the whale population, calculating the fitness, updating the whale positions, handling boundary conditions, and generating initial positions. In Step 2, the algorithm checks whether the maximum number of iterations has been reached. If not, it proceeds to Step 3, where a probability-based decision is made. Based on the value of P, the algorithm selects a behavior mode: if P≥ 0.5, the spiral position update is performed; if P< 0.5, it proceeds to Step 4. In Step 4, the algorithm determines the step coefficient based on the relative distance between the whale and the prey using the value of A. If $\left|A\right|\geq$ 1, the whale performs a search for prey; if $\left|A\right|<$ 1, the whale engages in shrinking surround behavior. Finally, the best solution is selected based on the optimal fitness value, and the process returns to Step 2 to continue the iterations until the stopping condition is met. The WOA flow is illustrated in Figure 10. The core functionality of the WOA is to identify an optimal set of control parameters that achieves a defined optimization objective. Inspired by the hunting behavior of humpback whales, the algorithm continuously updates its search position within a specified range, emphasizing extensive exploratory behavior initially. As the iterations progress and the search narrows, the WOA transitions to intensified local optimization. Based on these iterative optimization results, the WOA ultimately determines the torque distribution ratio between the motors.

## 4. Simulation and Experimental Results

In this study, the MATLAB/Simulink^®^ software is used to integrate the primary and secondary systems of a dual-motor electric vehicle. Both a pure simulation and HIL simulation are employed to test the RBC, GGS, and WOA control methods for the entire vehicle system. The simulation results are then analyzed and discussed accordingly.

### 4.1. GGS Grid Point Testing

In this study, the resolution of the GGS method is constrained by grid spacing and hardware limitations. If the interval between parameter grid points is too large, the calculated conditions may not precisely align with the points on the multi-dimensional grid. In such cases, the system uses built-in interpolation methods to obtain a solution. Although this reduces the computation time, the results may be suboptimal. Therefore, energy efficiency tests were conducted under the FTP-75 driving cycle using various GGS grid spacings to identify the most suitable grid design as a reference standard. As shown in Table 6, the average energy efficiency results over five FTP-75 simulations were recorded for grid spacings of 1, 0.5, 0.2, 0.1, 0.01, 0.005, and 0.001.

### 4.2. Parameter Adjustment Testing of Whale Optimization Algorithm

In this study, the parameters of the WOA were adjusted with respect to the count of iterations and the whale population size. Energy efficiency tests were conducted using the FTP-75 driving cycle, and the simulation results are presented in Table 7. It can be observed that the optimal (i.e., lowest) energy efficiency was achieved with 300 iterations and a whale population size of 50, as shown in Table 8. To explore the maximum simulation capacity of the hardware system, the iteration count was further increased from 300 to 400 to determine the highest number of iterations that could still be tested in real time.

### 4.3. Comparison of Vehicle Velocity Results

**Figure 11:**
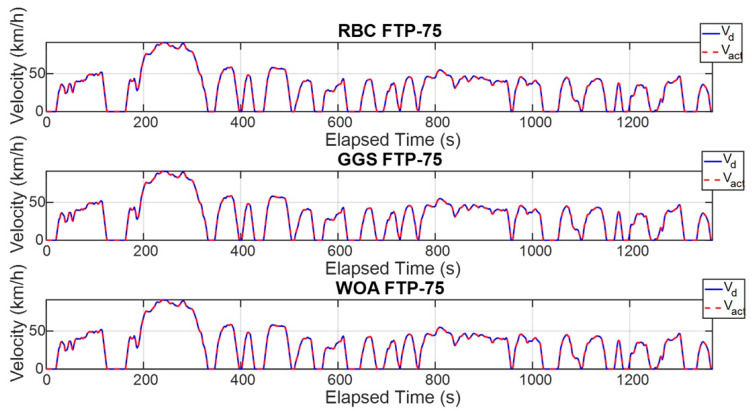
Vehicle velocity for a single FTP-75 driving cycle.

**Figure 12:**
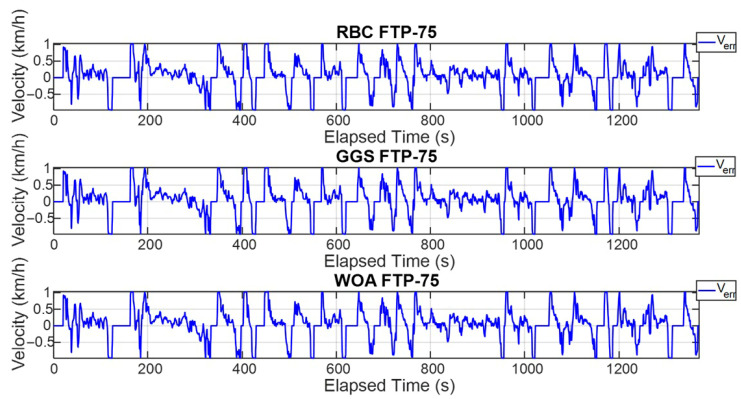
Vehicle velocity error for a single FTP-75 driving cycle.

**Figure 13:**
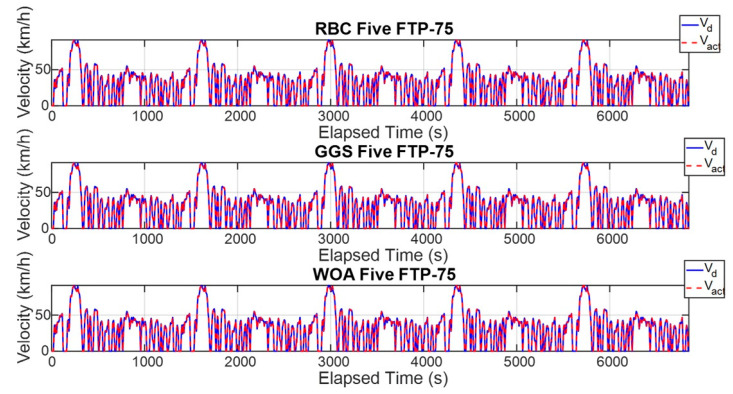
Vehicle velocity for five FTP-75 driving cycles.

**Figure 14:**
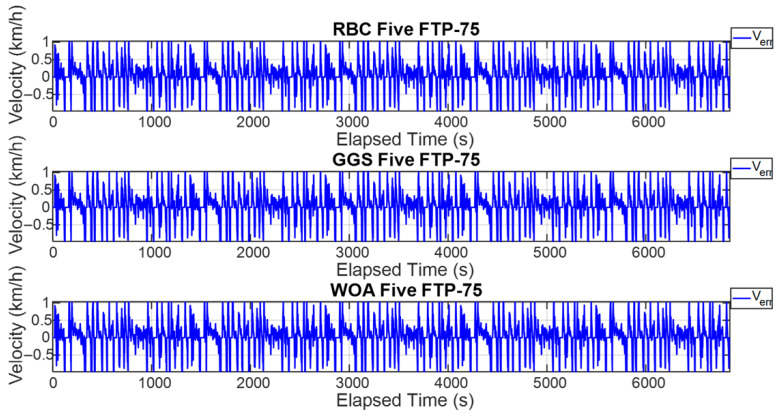
Vehicle velocity error for five FTP-75 driving cycles.

The FTP-75 driving cycle is mainly divided into two parts: the first part, from the start to the 505th second, is the cold-start phase, which primarily involves low- and medium-velocity driving patterns, including acceleration, cruising, and deceleration, with a maximum velocity of 91.25 km/h. The second part, from the 506th second to the 1370th second, is the hot-start phase, which features driving patterns similar to the cold-start phase, also including acceleration, cruising, and deceleration, with the same maximum velocity of 91.25 km/h. The energy management strategy determines the output torque of the dual power units based on the target vehicle velocity and the optimized power distribution ratio. To comprehensively verify whether the power distribution results of the energy management strategy can satisfy the required vehicle velocity, this study conducted both a single-cycle and five-cycle FTP-75 test. Figure 11 and Figure 12 show the vehicle velocity tracking and velocity error results for a single FTP-75 cycle. As observed from the two graphs, the dual power units provide sufficient power to reach the target velocity, with the velocity error controlled within ±1 km/h. However, according to Figure 13 and Figure 14, which present the vehicle velocity tracking and velocity error results over five FTP-75 cycles, it can be seen that, under the RBC, GGS, and WOA control strategies, the dual-motor system is capable of delivering sufficient power to achieve the target velocity, with the velocity errors maintained within ±2 km/h.

### 4.4. Torque Output Results Under Different Control Strategies

**Figure 15:**
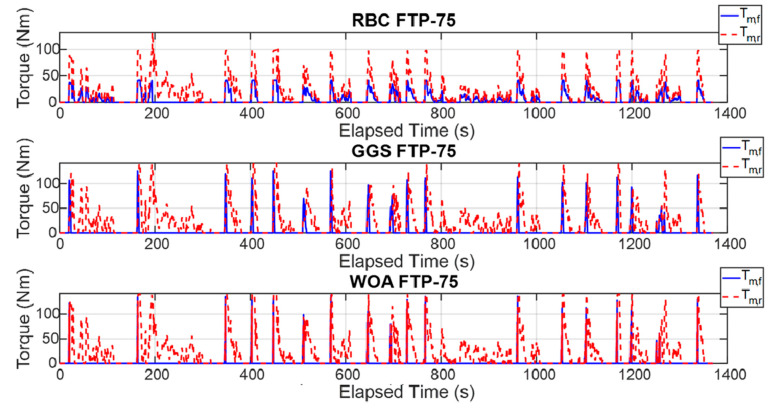
Torque output of the front- and rear-axis motors for a single FTP-75 driving cycle.

**Figure 16:**
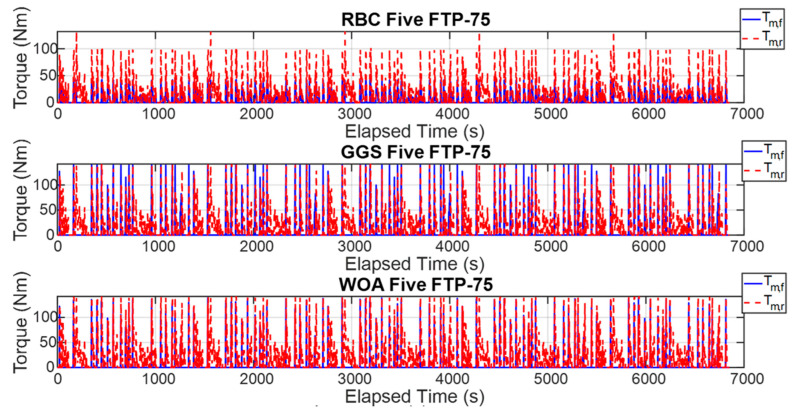
Torque output of the front- and rear-axis motors for five FTP-75 driving cycles.

The energy management strategy proposed in this study aims to achieve higher energy efficiency and improved computational efficiency by applying three control strategies to regulate the power output of the front- and rear-axle motors. Figure 15 illustrates the torque outputs of the front- and rear-axle motors during a single execution of the FTP-75 driving cycle, whereas Figure 16 presents these results over five consecutive FTP-75 driving cycles. According to the RBC strategy shown in Figure 15 and implemented based on the control logic defined in Table 3, the power distribution during a single FTP-75 driving cycle operates as follows: during vehicle acceleration, the front- and rear-axis motors output torque in a 30% to 70% distribution ratio. When the vehicle velocity exceeds 50 km/h, the demanded torque is primarily handled by the rear-axis motor. Under the GGS and WOA control strategies, the initial acceleration demanded torque is mainly provided by the front motor. However, during mid-range acceleration, the front motor ceases power output, and, once the velocity exceeds 50 km/h, the rear-axis motor becomes the primary source of torque. As shown in Figure 16, the five-cycle driving behavior closely follows the same trend observed in the single-cycle scenario. All three control strategies are capable of meeting the intended control design requirements.

### 4.5. HIL Output Results Under Different Control Strategies

**Figure 17:**
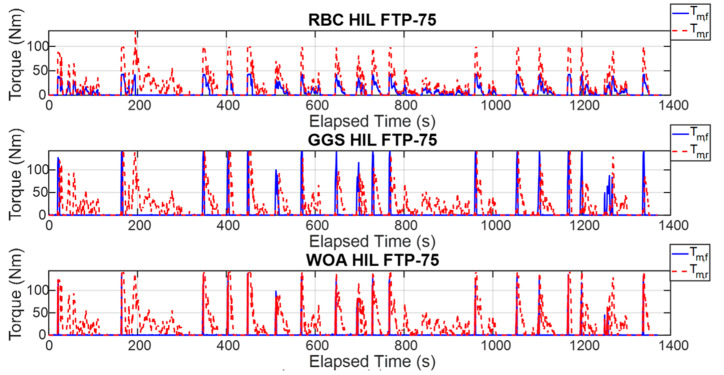
HIL output results under different control strategies for a single FTP-75 driving cycle.

**Figure 18:**
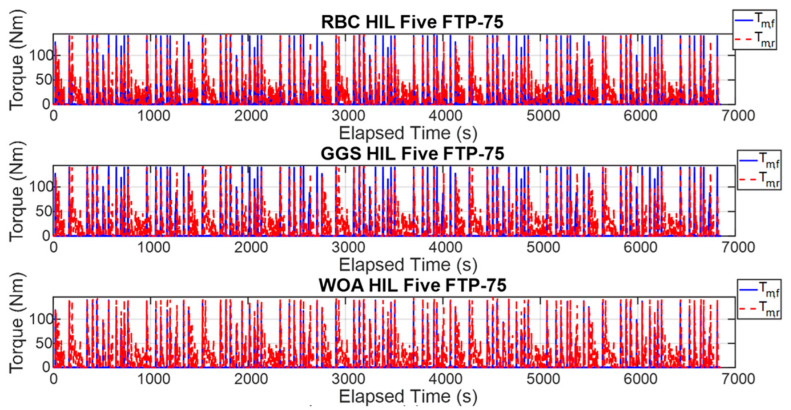
HIL output results under different control strategies for five FTP-75 driving cycles.

To validate the effectiveness of the proposed control strategies, this study employed HIL testing to verify the consistency between the dual-power model outputs and WOA computer simulation results. Figure 17 presents the motor torque output results from a single execution of each strategy for the front- and rear-axle motors, while Figure 18 shows the motor torque output results from five executions of each control strategy. As observed in Figure 17, under the RBC strategy, the torque output frequency of the front-axle motor is relatively low and concentrated primarily during specific acceleration phases. In contrast, the rear-axle motor consistently outputs torque throughout most of the operation, indicating that the rear motor primarily drives the vehicle. Examining the torque output results for the GGS and WOA strategies, the front-axle motor exhibits regular torque outputs during vehicle acceleration phases, demonstrating more synchronized output behavior between the front- and rear-axle motors under high-torque conditions. According to Figure 18, the torque outputs from the front- and rear-axle motors for both computer simulations and HIL tests demonstrate a certain degree of similarity. The high frequency of front- and rear-axle coordination across all three control strategies ensures effective utilization. However, discrepancies are apparent in the HIL environment due to constraints such as noise and system overheating. While similarities remain evident between the simulation and HIL torque outputs, noticeable oscillations exist, likely caused by signal scaling, which introduces differences between the transmitted and original signals, thereby generating errors.

### 4.6. Energy Efficiency Results Under Different Control Strategies

The energy efficiency of the entire vehicle, simulated under various control strategies, serves as a benchmark for comparison. The detailed results are shown in Table 8 and Table 9. These tables, respectively, present the energy efficiency outcomes under three different control strategies (RBC, GGS, and WOA), comparing both the pure simulation and HIL scenarios. According to the data analysis from Table 9 and Table 10, it is evident that both the GGS and WOA control strategies significantly improve the energy efficiency. Specifically, the GGS strategy demonstrates slightly better energy performance than the WOA strategy. However, the WOA strategy still exhibits outstanding performance, with results closely aligning with those of GGS. Compared to the RBC strategy, the GGS and WOA strategies improve the energy efficiency by 9.1% and 8.9%, respectively. Further analysis of the HIL testing results shows that the WOA-based energy management strategy not only performs well in computer simulations but also demonstrates high feasibility in real-world hardware environments. The HIL test results indicate that, compared to the RBC strategy, the GGS and WOA strategies improve the energy efficiency by 4.2% and 3.8%, respectively. Although the GGS strategy achieves slightly better energy efficiency, the WOA strategy proves to be a highly promising alternative for the practical implementation of efficient energy distribution. The results demonstrate that the GGS method exhibits comprehensiveness by systematically and exhaustively exploring all potential solutions, thus minimizing the risk of missing the global optimum. Its thorough coverage of the search space enhances the reliability and credibility of the obtained solutions. However, GGS is computationally intensive, requiring extensive resources and significantly longer computation times, thereby limiting its efficiency for high-dimensional or complex problems. Consequently, it is less adaptable to rapidly changing, real-time dynamic conditions. In this research, it nearly reached full computational capacity during the HIL simulations. In contrast, the WOA efficiently computes near-optimal solutions with substantially fewer computational resources. It is particularly well suited for high-dimensional and complex optimization problems, adeptly handling non-linear and multi-variable scenarios. Furthermore, the WOA demonstrates superior responsiveness in dynamic real-time environments. This study indicates that it achieves HIL simulations at approximately half of the computational load required by GGS. Nevertheless, the stability of the optimal solutions from the WOA is relatively lower, with the results potentially varying slightly between executions. Thus, averaging the results from multiple runs is necessary to ensure reliability.

### 4.7. Regenerative Braking Results Under Different Control Strategies

**Figure 19:**
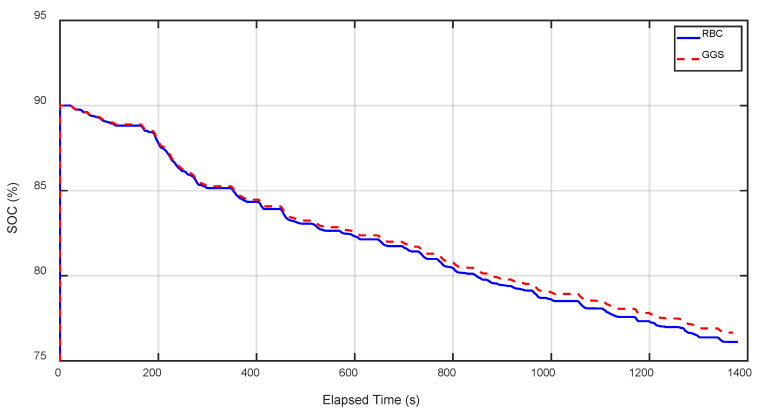
SOC output results under different control strategies for five FTP-75 driving cycles (no regenerative braking).

**Figure 20:**
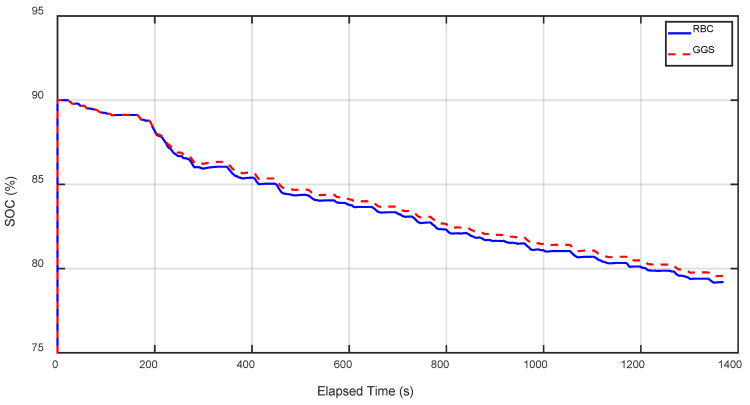
SOC output results under different control strategies for five FTP-75 driving cycles (regenerative braking).

In Figure 19 and Figure 20, the battery SOC comparison results for RBC and GGS under the HIL simulation with and without regenerative braking are presented. Both RBC and GGS significantly reduced the battery SOC consumption when regenerative braking was incorporated. Table 11 and Table 12, respectively, show the SOC comparison results for RBC and GGS under the pure simulation and HIL simulation with the addition of regenerative braking. Data analysis from Table 11 and Table 12 indicates that adding regenerative braking notably improves the energy efficiency. Specifically, compared to the pure RBC strategy, the RBC and GGS strategies with regenerative braking improved the energy efficiency by 26.8% and 30.67%, respectively. Further analysis of the HIL test results shows that, compared to the pure RBC strategy, the RBC and GGS strategies with regenerative braking improved the energy efficiency by 26.1% and 29.4%, respectively. The proposed regenerative braking strategy covers torque distribution during the deceleration and braking phases, ensuring that both the front and rear motors effectively contribute to energy recovery while maintaining vehicle stability.

## 5. Conclusions

This study established a simulation platform using MATLAB/Simulink^®^ to model a real-world Tesla Model X vehicle equipped with front- and rear-axle motor systems. The specifications and characteristic curves of each power source system were developed, and the following conclusions were drawn.

➢ Dual-Motor Electric Vehicle Model Development: A physics-based control model was developed using existing Tesla Model X vehicle parameters. Speed-tracking simulations were performed under various control strategies. The constructed model comprises sub-models for the driving cycle, driver behavior, electric motor dynamics, lithium battery characteristics, vehicle dynamics, and energy management system, ensuring the reliable and stable operation of the front- and rear-axle motor systems in practical applications.
➢ Application of Energy Control Strategies: Based on the dual-motor system simulation platform developed in MATLAB/Simulink^®^, this study implemented and tested three different control strategies: RBC, GGS, and WOA.
➢ Validation via Pure Simulation and HIL Simulation: A closed-loop real-time simulation platform was established using an Arduino DUE microcontroller and a TI C2000 microcontroller in series. This platform was used to verify the WOA-based energy management system. Real-time computation was carried out in a HIL environment, and the results were compared with those from the pure simulation to assess the consistency.
➢ Validation Under FTP-75 Driving Cycle:
  - Pure Simulation: Compared with the RBC, the GGS and WOA control strategies improved the energy efficiency by 9.1% and 8.9%, respectively.
  - HIL Simulation: Energy efficiency improvements of 4.2% and 3.8% were achieved using GGS and WOA, respectively, compared to the RBC.
  - Compared to the pure RBC strategy, the RBC and GGS strategies incorporating regenerative braking achieved energy efficiency improvements of 26.1% and 29.4%, respectively.

## Figures and Tables

**Table 1:** Specifications of the electric vehicle.

Item		Specification
Drive Motor	Type	Permanent magnet synchronous
	Maximum Output Power	250 kW * 2
	Maximum Output Torque	250 Nm@4750 rpm
Energy Storage Battery	Type	Lithium-ion battery
	Rated Voltage	432 V
	Maximum Capacity	64.58 kWh
Vehicle Parameters	Vehicle Mass	2373 kg
	Aerodynamic Drag Coefficient	0.24
	Frontal Area	3.5 m^2^
	Tire Radius	0.275 m
	Rolling Resistance	0.01
	Final Drive Ratio	9.78
	Road Friction Coefficient	0.95

**Table 2:** Signal conversion of the Arduino DUE and Ti C2000 microcontrollers.

Range	Arduino DUE		Conversion	Ti C2000	
--	Digital	Analog	--	Digital	Analog
Min.	0	0.56 V	DAC	0	0 V
Max.	4095	2.76 V		4095	3 V
Min.	0	0 V	ADC	0	0 V
Max.	4095	3.3 V		4095	3 V

**Table 3:** Power distribution mode of the RBC.

Mode	Condition	Action
System Ready	$T_{d}=0$ Nm	$T_{m_{,f}}=0\;\mathrm{Nm};\;T_{m,r}=0$ Nm
Low Load	$T_{d}>1$ Nm	$T_{m_{,f}}=0.3\times T_{d};\;T_{m,r}=0.7\times T_{d}$
High Load	$50\;\mathrm{km}/h\;<V_{act}$	$T_{m_{,f}}=0\;\mathrm{Nm};\;T_{m,r}=T_{d}$
Safety	$T_{d}$ = 0 Nm and SOC = 0	$T_{m_{,f}}=0\;\mathrm{Nm};\;T_{m,r}=0$ Nm

**Table 4:** The relevant parameters of the GGS for the front- and rear-axis motors.

Parameter	Value
$T_{d}$	0:50:250
$N_{m}$	0:500:13,000
α	0:0.1:1

**Table 5:** Parameter settings of the WOA.

Parameter	Value
$W_{N}$	50
$n_{max}$	340
r	[0, 1] Random Value
b	1
j	[−1, 1] Random Value
Searching Space of $T_{m}$	[0, $T_{m,max}(N_{m}$)]

**Table 6:** Energy efficiency comparison of grid sizes for GGS.

Grid Size	Energy Efficiency (km/kWh)
1	3.94008
0.5	3.94126
0.2	3.94221
0.1	3.94261
0.01	3.94361
0.005	N/A
0.001	N/A

**Table 7:** Comparison of energy efficiency for different WOA iteration counts and whale population sizes.

	Count	10	100	200	300	400
Size						
10		3.87261	3.87987	3.88092	3.88101	N/A
25		3.87666	3.88024	3.88093	3.88101	N/A
50		3.87822	3.88045	3.88097	3.88102	N/A
100		3.87824	3.88045	3.88097	3.88102	N/A
150		3.87824	3.88045	3.88097	3.88102	N/A

**Table 8:** Energy efficiency comparison of WOA with 300 and 400 iterations.

	Count	300	320	340	360	380
Size						
10		3.88102	3.88113	3.88129	N/A	N/A

**Table 9:** Energy efficiency comparison of the pure simulation.

Strategy	Energy Efficiency (km/kWh)	Improvement Rate (%)
RBC	3.5585	--
GGS	3.9188	9.1
WOA	3.9063	8.9

**Table 10:** Energy efficiency comparison of the HIL simulation.

Strategy	Energy Efficiency (km/kWh)	Improvement Rate (%)
RBC	3.7510	--
GGS	3.9137	4.2
WOA	3.8991	3.8

**Table 11:** Energy efficiency comparison of regenerative braking in pure simulation.

Strategy	Energy Efficiency (km/kWh)	Improvement Rate (%)
RBC	3.5585	--
RBC + regenerative braking	4.8633	26.8
GGS	3.9188	9.19
GGS + regenerative braking	5.1326	30.67

**Table 12:** Energy efficiency comparison of regenerative braking in HIL simulation.

Strategy	Energy Efficiency (km/kWh)	Improvement Rate (%)
RBC	3.7510	--
RBC + regenerative braking	5.0758	26.1
GGS	3.9137	4.16
GGS + regenerative braking	5.3131	29.4

## Data Availability

The original contributions presented in this study are included in the article. Further inquiries can be directed to the corresponding author.

## Nomenclature

α	power distribution ratio of the first gear
ω	represents the penalty value;
μ	rolling resistance coefficient
$\rho_{a}$	air density
$\eta_{b}$	efficiency of battery charge and discharge
$\eta_{f}$	overall efficiency of the final drive
$\eta_{m}$	efficiency of the drive motor
$\eta_{m,f}$	efficiency of the front-axis motor
$\eta_{m,r}$	efficiency of the rear-axis motor
A	step coefficient
$A_{f}$	frontal area of the EV
a	decreases linearly from 2 to 0 during the iteration process
b	constant that defines the shape of the spiral bubble net
C	weighting coefficient
$C_{d}$	drag coefficient
$D^{\prime }$	position vector between the whale and a randomly selected prey
$D^{\prime \prime }$	spatial vector between the whale and the current best prey position
${\dot{E}}_{m,f}$	input power of the front-axis motor
${\dot{E}}_{m,r}$	input power of the rear-axis motor
$F_{brk}$	braking force
g	gravitational acceleration
$I_{b}$	charge and discharge current of the battery
J	optimal objective function
j	random number within [−1, 1]
$K_{I}$	integral gain of the PI controller
$K_{P}$	proportional gain of the PI controller
${Low}_{bound}$	lower bound of the control variable being searched
$m_{EV}$	total vehicle mass
$N_{m}$	wheel speed of the drive motor
$n_{max}$	maximum number of iterations
$n_{t}$	current iteration number
P	probability
$P_{b}$	discharge power of the battery
$Q_{rated}$	rated capacity of the lithium battery
$R_{b}$	equivalent internal resistance of the lithium battery gradient resistance
$R_{w}$	wheel radius
r	random vector within the range [0, 1]
${SOC}_{b}$	state of charge for lithium battery
${SOC}_{int}$	state of charge initial value for lithium battery
$T_{d}$	total demand torque
$T_{f}$	output torque of the final drive
$T_{m}$	output torque of the drive motor
$T_{m,f}$	output torque of the front-axis motor
$T_{m,r}$	output torque of the rear-axis motor
t	time or current number of iterations
$t_{b}$	temperature of the battery
${Up}_{bound}$	upper bound of the control variable being searched
${Variable}_{size}$	dimensional size of the control variable
$V_{b}$	voltage of the lithium battery
$V_{v}$	vehicle velocity
$V_{OC}$	open-circuit voltage of the lithium battery
$V_{err}$	error between the demand vehicle velocity and the actual vehicle velocity
$W_{N}$	population size of whales
X	current position
$X^{*}$	position of the current best solution
$X_{rand}$	current random position of the whale population
